# Supplementary material for: Synthetic asters as elastic and radial skeletons
Source: Nat Commun. 2019 Oct 31;10:4954. doi: 10.1038/s41467-019-13009-4 (PMC6823511; doi:10.1038/s41467-019-13009-4)
Supplement: Supplementary file 1 — Supplymentary Information [file 41467_2019_13009_MOESM1_ESM.pdf]

Supplementary Information

Synthetic asters as elastic and radial skeletons

Qingqiao Xie,[1] Xixi Chen,[2] Tianli Wu,[2] Tiankuo Wang,[3] Yi Cao,[3] Steve Granick,[4,5] Yuchao Li,[2,*] and Lingxiang Jiang[1,4,*]

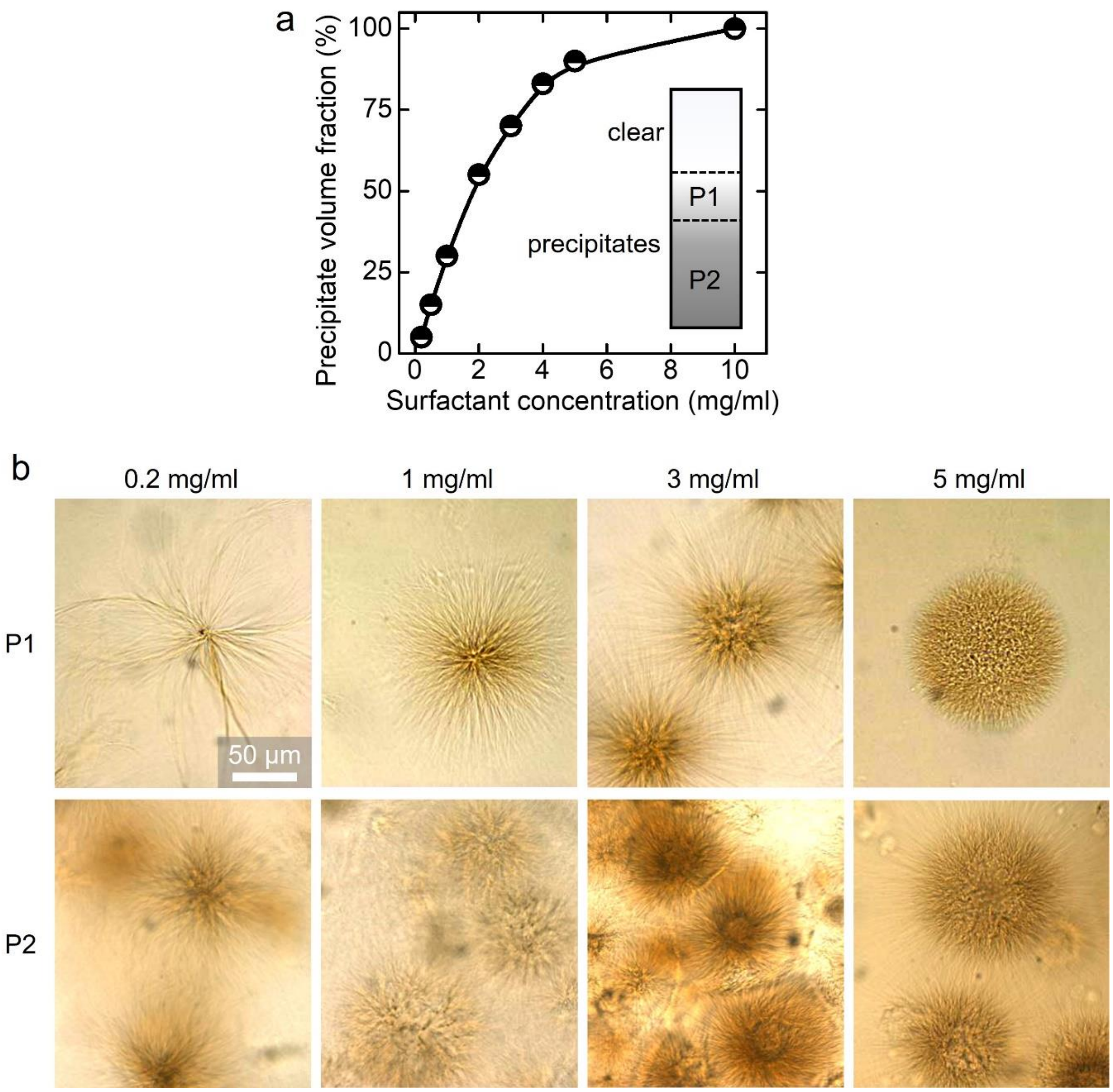

Supplementary Figure 1. Effect of surfactant concentration on aster morphology. a, Upon cooling, the suspension separate into a clear, upper phase and a turbid, lower phase. The lower phase gradually expands with higher surfactant concentrations and finally fills up all the volume. The lower phase can be roughly divided into P1 close to the phase boundary and P2 at the vial bottom. **b**, Asters formed with different surfactant concentrations and in different vial regions are inspected by optical microscopy. With higher concentration, ribbon density increases, aster core grows significantly, but the overall size remains roughly constant. Comparing P1 and P2 region, we notice that the asters are loosely packed with lighter ribbons in P1.

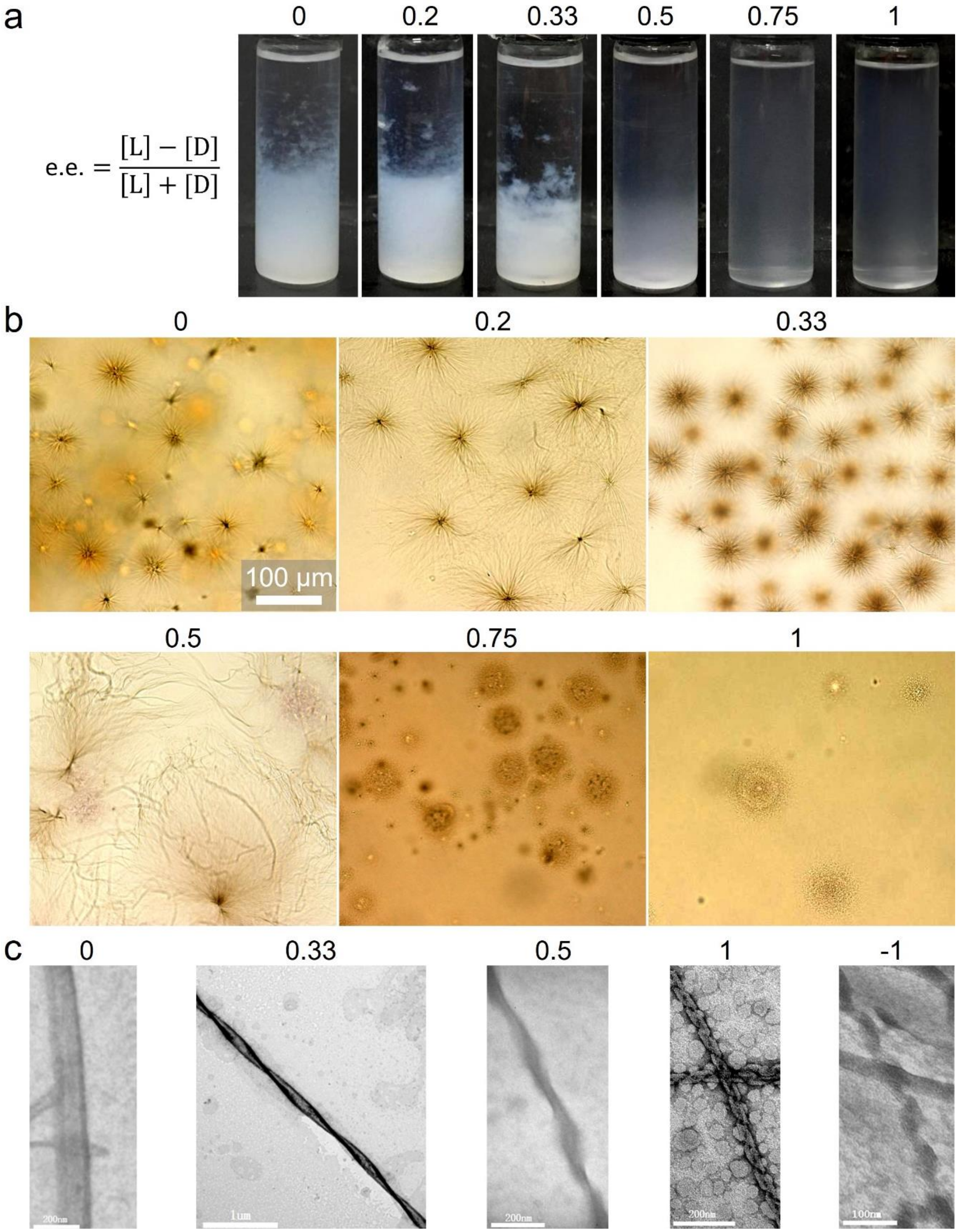

Supplementary Figure 2. Effect of counterion ratio on aster morphology. a, Enantiomeric excess, e.e., is defined by the concentrations of D- and L-tartrate. In the main text, we focused on e.e. = 0.33. Macroscopic pictures are listed for samples of a constant surfactant concentration of 1 mg/ml but different e.e. value. At e.e. = 0.75 and 1, there are small semitransparent droplets that barely phase separate. **b**, Asters form at e.e. = 0 to 0.5, although their ribbons are remarkably soft and floppy at e.e. = 0.5. No aster

is observed at e.e. = 0.75 and 1 or -1. Instead, we recognize these structures as cores (spherical networks of nodes connected by ribbons, the ribbons cannot be seen by OM but can be seen by TEM) that do not transform into asters. We do not yet understand why they stay as cores. c, Helicity of the ribbons is regulated by e.e., as reported in Ref. 21.

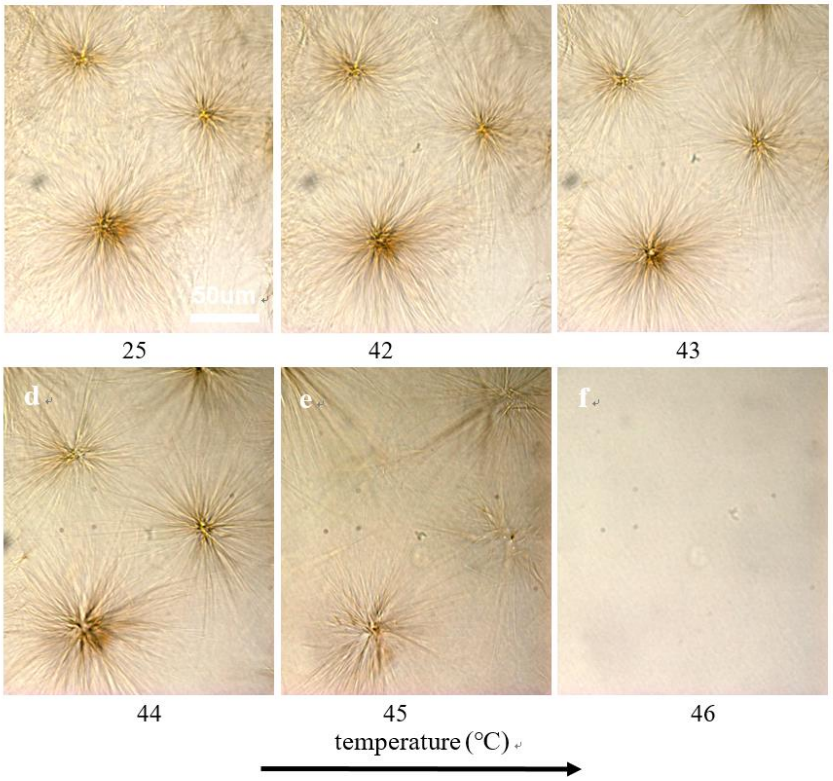

Supplementary Figure 3. Melting of asters upon heating above the Krafft point.

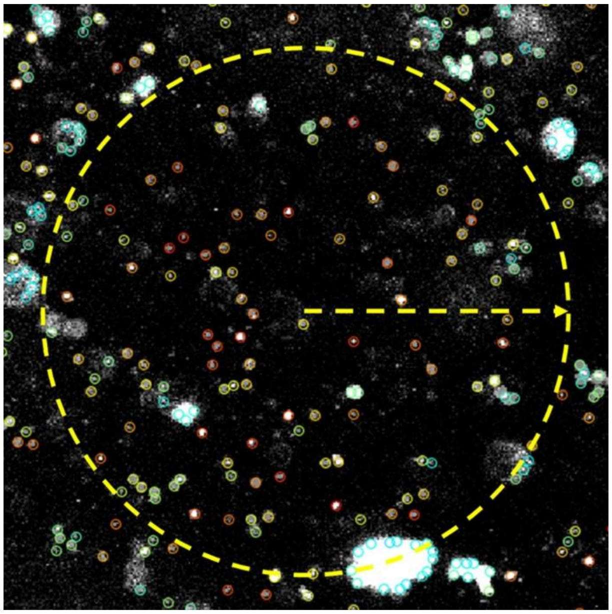

Supplementary Figure 4. Fluorescent, type-B particles scattered in an aster are tracked by TrackMate. This image is the fluorescence channel in gray scale overlaid with aster outer rim (yellow circle) and tracking circles (color denoting contrast).

Supplementary Table 1. Comparison between the biological and synthetic asters and previously reported core-ray particles.*

		Structure					Property		Function	
	Ref.	Size (μm)	Ray den.	Heli.	Pola.	Elasticity	Dyn.	Pos.	Act.
Microtubule asters	2, 4	5 to 100	moderate	Y	Y	semiflexible	Y	Y	Y
Synthetic asters (this work)		50 to 120	moderate	Y	N	semiflexible	N	Y	N
$BaCO_3/SiO_2$ hemi-asters	10	~50	moderate	Y	N	rigid	N	NA	N
ZnO Hedgehog particles	15	~1	moderate	N	N	rigid	N	NA	N
SiO_2 spiky particles	11	~1	moderate	N	N	rigid	N	NA	N
Protein spherulites	14	20 to 100	compact	Y	N	rigid	N	N	N
Malonamide spherulites	13	~100	compact	N	N	rigid	N	N	N
Coacervate-based asters	18	~50	moderate	Y	N	soft	N	NA	N
$Ca_3(PO_4)_2$-polymer particles	9	0.2	low	N	N	NA	N	NA	N

*Abbreviations: Ref. = reference; Ray den. = ray density; Heli. = helicity; Pola. = polarity; Dyn. = dynamic nature; Pos. = positioning capability; Act. = active transportation; Y = yes; N = no; NA = not available.

The biological asters feature helical, polar, semiflexible microtubules radiating from a center at a moderate density. The microtubules are semiflexible filaments with a persistence length ~ 4 mm, which can sustain a certain stress and deform to an extent. The microtubules are dynamic filaments that constantly undergo assembly/disassembly by consuming ATP. The asters are crucial in subcellular positioning and active transportation.

In comparison, the synthetic ones can be roughly categorized into inorganic, organic, and hybrid particles. They are markedly different in size, ray density, elasticity, and functionality. Many synthetic ones are made of rigid rays that cannot deform under a biologically relevant force; the coacervate-based asters are too soft to sustain certain stress. Two spherulites are too compact to accommodate any particles

precluding their application in particle positioning. The $BaCO_3/SiO_2$ structures were fabricated by "microsculpturing" on a substrate, so they are attached to the substrate and are only hemi-asters. The $Ca_3(PO_4)_2$-polymer hybrid particles are 200 nm in size too small to position other particles. Overall, the main advance in this work is that the current asters feature cell-like elasticity and capability in particle positioning, whereas the previous core-ray structures fall short in these two aspects. Notably, all the synthetic asters cannot parallel the biological ones in terms of polarity, dynamic nature, and active transportation yet.
